# Tort Reform Is Associated with Significant Increases in Texas Physicians Relative to the Texas Population

**DOI:** 10.1007/s11605-012-2013-4

**Published:** 2012-09-29

**Authors:** Ronald M. Stewart, Molly West, Richard Schirmer, Kenneth R. Sirinek

**Affiliations:** 1 Department of Surgery, University of Texas Health Science Center at San Antonio, San Antonio, TX USA; 2 University Hospital, San Antonio, TX USA; 3 Texas Hospital Association, Austin, TX USA; 4 Jocelyn and Joe Straus Endowed Chair for Trauma Research, Department of Surgery, University of Texas Health Science Center at San Antonio, 7703 Floyd Curl Drive, San Antonio, TX 78229 USA

**Keywords:** Licensure, medical, Liability, legal, Health care reform, Tort reform, Insurance, liability, Malpractice, Jurisprudence, Litigation, Risk, Health policy, Healthcare access

## Abstract

**Introduction:**

Texas implemented comprehensive tort reform in 2003. We hypothesized that tort reform was followed by a significant increase of physicians practicing in Texas.

**Methods:**

To test this hypothesis, we compared the rate of physician growth prior to and following tort reform, and the number of licensed physicians and physicians per 100,000.

**Results:**

Comparing before and after tort reform, the rate of increase in Texas physicians per 100,000 population increased significantly (*p* < 0.01). From 2002 to 2012, the Texas population increased 21 %. The number of actively practicing Texas physicians increased by 15,611 a 44 % increase (46 % metro areas vs. 9 % non-metro areas), an increase of 30 physicians per 100,000 population (*p* < 0.01). Non-metropolitan Texas had a net increase of 215 physicians; however, there was no change in the number of physicians per 100,000. Examining the data by trauma service areas (TSAs), 20 of 22 TSAs had an increase in both number of physicians and physicians per capita, five greater than 50 %.

**Conclusions:**

The post-tort reform period in Texas was associated with a significantly increased growth rate of physicians relative to the Texas population. Tort reform, as implemented in Texas, provides a needed framework for improving access to health care.

## Introduction

The economic impact of rising medical malpractice premiums and the cost of associated litigation had reached a crisis level by 2002 in many regions of the United States, including Texas.^1^ The financial and emotional impact of malpractice claims were driving physicians and surgeons away from high-risk specialties as well as from high-risk, litigious, geographic environments.^2^

In 2003, the Texas state legislature enacted comprehensive tort reform laws that included a cap on noneconomic damages in most medical malpractice cases at $250,000.^3^ Texas voters subsequently approved a state constitutional amendment supporting this legislation.^4^ Multiple reports have documented dramatic decreases in the cost of medical malpractice premiums across the state for all specialties, along with a decrease in the incidence of medical malpractice claims and lawsuits.^1^,^5^–^8^ In our own practice, we witnessed a 5-fold decrease in the risk of general surgical malpractice lawsuits and a 55 % decrease in premiums after the implementation of comprehensive tort reform in Texas in 2003.^7^ Less evident is whether tort reform achieved its intended goal of increasing access to health care for Texas citizens.^8^,^9^

We hypothesized that comprehensive tort reform led to significant increases in the total number of physicians practicing in Texas and the number of physicians relative to the Texas population. To test this hypothesis, we compared the number of licensed physicians by both specialty and geographic location before and after the implementation of comprehensive tort reform.

## Methods

### Data Sources

The study was performed using publicly accessible data from the Texas Medical Board (TMB), the United States Census Bureau/Texas State Library and Archives Commission and the Texas Department of State Health Services.^10^–^12^ The TMB is the statutorily directed authority that regulates the practice of medicine in Texas. The TMB maintains detailed data with respect to physician demographics, status of practice, location of practice by county, complaints, compliance, litigation and enforcement.^10^ These data sets are electronically accessible to the public from January 1999 to December 2010. The TMB supplied archival data for 1995–1998. The TMB’s statutory authority is based on 18 chapters of the Occupations Code. Agency statutes have undergone major revisions in recent legislative sessions. In June 2003, the 78th Texas Legislature passed comprehensive tort reform in House Bill 4, which became effective September 1, 2003.^3^ During the same legislative session, Senate Bill 104 provided statutory reinforcement and strengthening of the TMB along with additional funding.^13^

### Description of Geographic Areas

Texas consists of 254 separate counties encompassing 268,581 square miles. In 2011, the estimated Texas population was 25,674,681. Texas counties range in size from a minimum of 127 square miles to a maximum of 6,184 square miles. County populations range from a minimum of 65 people to a maximum of 4.3 million people. County population density in these counties ranges from a minimum of 0.1 person per square mile to a maximum of 2,851 persons per square mile. Texas consists of 25 distinct metropolitan service areas (MSAs). Eighty-eight percent of Texans reside in these MSAs. Texas consists of 22 trauma service areas (TSAs). These TSAs were defined in 1989, with geographic divisions along traditional, regional, medical practice referral lines. Each TSA is large enough to support at least one regional trauma center.

### Hospital Survey

In July 2008, the Texas Hospital Association surveyed hospitals and health systems to measure the impact of medical liability reform (http://www.tha.org/HealthCareProviders/Issues/TortReform/Hospitals%20Reap%20Benefits%20of%20Tort%20Reform.pdf). The survey asked five questions: (1) What impact has decreased liability insurance cost had on operations or provision of services? (2) Has the hospital been able to expand services based on decreased hospital liability expense?; (3) Has the hospital been able to expand emergency or specialized services (trauma, surgery, etc.) due to a larger number of physicians willing to take call or expand their practice? (4) If your facility has maintained or expanded emergency or specialized services to what extent has declining physician liability expense or a more favorable liability climate in Texas contributed? (5) Since September 2003 has your facility found it easier to recruit physicians?

### Data Analysis

TMB data tables, with respect to number of physicians practicing in Texas, and the number and specialty of physicians practicing in each county were retrieved. These data sets were translated into an electronic spreadsheet, Microsoft Excel**®** 2011 for Macintosh.

Actively practicing, licensed physicians in Texas were divided into broad specialty categories: (1) all physicians; (2) primary care physicians (PCPs) (emergency medicine, family medicine, internal medicine, general practice, pediatrics, geriatrics, general preventive medicine, and obstetrics and gynecology); (3) obstetrics and gynecology; and (4) all surgical specialties.

Comprehensive tort reform in Texas was implemented mid-year in 2003. The two periods prior to (January 1995–December 2002) and following 2003 (January 2004–December 2012) were used as pre-tort reform and post-tort reform periods.

Detailed specialty and demographic analysis was performed comparing the year immediately prior to tort reform, January 2002 to the present time, January 2012. This analysis was done using unadjusted data, data normalized per 100,000 population and data normalized per square mile. These data were analyzed by four geographic subdivisions: the entire state, by TSA, by MSA, and by individual county.

Nonparametric data were analyzed using a chi-square with Yates’ correction for nominal variables. Relative risk is displayed with 95 % CI. Statistical analyses were performed using SAS Version 9.3 for Windows (SAS Institute, Cary, NC), AnalystSoft, StatPlus:mac^**®**^ — statistical analysis program for Mac OS v. 2009, Microsoft Excel^**®**^, GraphPad Prism for Windows^**®**^. Differences were considered statistically significant if the *p* value was <0.05. For repeated measures, the Bonferroni correction was employed to reduce the possibility of false positive statistical associations. The change and rate of change in physicians per 100,000 in the pre-tort period (1995–2002) and post-tort period were analyzed using three different methods: chi square (sum of repeated annual measures pre-tort and post-tort reform), a linear regression model, and a repeated-measures Poisson model.

## Results

**Fig. 1:**
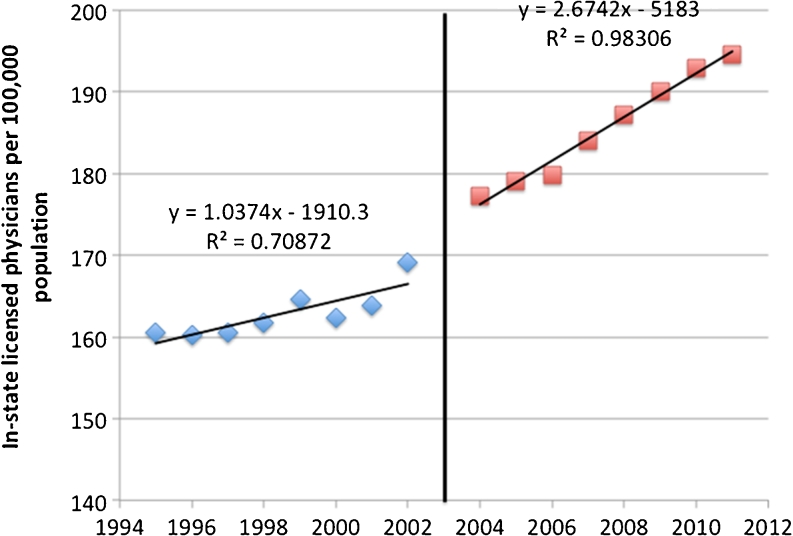
Total number of licensed physicians per 100,0000 residents before and after tort reform

When comparing the period after tort reform (2004–2012) to the period before tort reform (1995–2002), the rate of increase in Texas physicians per 100,000 Texas residents was significantly greater (*p* < 0.01 by chi square and linear regression) (Fig. 1). Based on the repeated measures Poisson model, the ratio of the number of physicians per resident per year increased by year and with tort reform (*p* < 0.001). Prior to tort reform, the increase per year was 0.854 %, 99 % CI (0.854 %, 0.854 %) and after tort reform the increase per year grew by 69 % to 1.45 % (1.45 %, 1.45) and the percentage increase was significantly different from zero both before (*p* < 0.001) and after (*p* < 0.001) tort reform.

The absolute growth in Texas physicians relative to absolute growth in the Texas population before and after tort reform demonstrates a 2-fold greater growth in the post-tort reform period compared to the pre-tort reform period.

A detailed comparison between 2002 and 2012 demonstrates the ramifications of these differing growth rates (Table 1: Texas Now and Immediately Prior to Tort Reform and Table 2: The Change from 2002 to 2012 by MSA). Table 1 summarizes the demographic changes from just prior to tort reform to 8.5 years following tort reform. From 2002 to 2012, the Texas population increased 21 %, with 88 % of Texans residing in a metropolitan area. Over this time period, Texas metropolitan areas increased in size disproportionately to non-metropolitan areas (23 % vs. 8 % increase in population, respectively). The number of Texas physicians increased by 15,611 (44 % increase with a 46 % increase in metro areas vs. a 9 % increase in non-metro areas). This absolute change led to an increase of 30 physicians per 100,000 population (19 % increase, *p* < 0.01). The non-metropolitan areas of Texas had a net increase of 215 physicians; however, there was no change in the number of physicians per capita in these non-metropolitan areas. Twenty-three of 25 MSAs had both an increase in the absolute number of physicians and an increase in physicians relative to the population being served. Twelve of these increases were statistically significant without Bonferroni correction (10 with Bonferroni correction). In general, the larger MSAs in central Texas had the greatest increase in physicians per capita. No MSA had a statistically significant decrease in the number physicians or physicians/100,000 population.

**Table 1:** Texas population and licensed actively practicing physicians in 2002 and 2012

Area Name	2002 Population	2012 Population	2002 Physicians	2012 Physicians	2002 Physicians per 100,000	2012 Physicians per 100,000
Texas	21,779,893	26,403,743	35,606	51,217	163	194
Metropolitan	18,831,827	23,213,579	33,124	48,520	176	209
Non-metropolitan	2,948,066	3,190,164	2,482	2,697	84	85
Abilene MSA	159,356	167,500	283	396	178	236
Amarillo MSA	232,215	261,345	473	607	204	232
Austin–Round Rock MSA	1,332,367	1,818,740	2,372	3,924	178	216
Beaumont–Port Arthur MSA	386,433	379,176	599	595	155	157
Brownsville–Harlingen MSA	356,745	433,449	427	530	120	122
College Station–Bryan MSA	193,758	216,544	325	450	168	208
Corpus Christi MSA	405,972	425,814	779	860	192	202
Dallas–Fort Worth–Arlington MSA	5,482,761	6,955,794	9,311	14,278	170	205
El Paso MSA	699,557	791,317	855	1,191	122	151
Houston-Sugar Land-Baytown MSA	4,967,350	6,280,138	9,261	13,985	186	223
Killeen–Temple–Fort Hood MSA	340,234	407,477	663	1,154	195	283
Laredo MSA	208,605	270,381	175	213	84	79
Longview MSA	197,186	215,359	291	365	148	169
Lubbock MSA	254,215	277,682	700	833	275	300
McAllen–Edinburg–Mission MSA	615,343	842,344	607	853	99	101
Midland MSA	117,298	133,004	175	208	149	156
Odessa MSA	122,926	135,331	185	252	150	186
San Angelo MSA	105,808	105,335	212	270	200	256
San Antonio MSA	1,782,686	2,156,984	3,602	5,285	202	245
Sherman–Denison MSA	114,545	123,128	152	244	133	198
Texarkana MSA	91,178	93,477	222	248	243	265
Tyler MSA	181,819	215,243	564	757	310	352
Victoria MSA	113,205	121,587	229	228	202	188
Waco MSA	217,826	238,787	383	497	176	208
Wichita Falls MSA	152,439	147,643	279	297	183	201

**Table 2:** Change by geographic area

	Population change	% population change	Net physician change	% net physician change	Change in physicians per 100,000	% change physicians per capita	*p* (Bonferroni correction)
Total Texas	+4,623,850	21 %	+15,611	44 %	+30	19 %	<0.01 (<0.01)
Total metropolitan	+4,381,752	23 %	+15,396	46 %	+33	19 %	<0.01 (<0.01)
Total Non-metropolitan	+242,098	8 %	+215	9 %	0	0 %	0.88 (NS)
Abilene MSA	+8,144	5 %	+113	40 %	+59	33 %	<0.01 (<0.01)
Amarillo MSA	+29,130	13 %	+134	28 %	+29	14 %	0.03 (NS)
Austin–Round Rock MSA	+486,373	37 %	+1,552	65 %	+38	21 %	<0.01 (<0.01)
Beaumont–Port Arthur MSA	−7,257	−2 %	−4	−1 %	+2	1 %	0.83 (NS)
Brownsville–Harlingen MSA	+76,704	22 %	+103	24 %	+3	2 %	0.74 (NS)
College Station–Bryan MSA	+22,786	12 %	+125	38 %	+40	24 %	<0.01 (0.09)
Corpus Christi MSA	+19,842	5 %	+81	10 %	+10	5 %	0.3 (NS)
Dallas–Fort Worth–Arlington MSA	+1,473,033	27 %	+4,967	53 %	+35	21 %	<0.01 (<0.01)
El Paso MSA	+91,760	13 %	+336	39 %	+28	23 %	<0.01 (<0.01)
Houston–Sugar Land–Baytown MSA	+1,312,788	26 %	+4,724	51 %	+36	19 %	<0.01 (<0.01)
Killeen–Temple–Fort Hood MSA	+67,243	20 %	+491	74 %	+88	45 %	<0.01 (<0.01)
Laredo MSA	+61,776	30 %	+38	22 %	−5	−6 %	0.44 (NS)
Longview MSA	+18,173	9 %	+74	25 %	+22	15 %	0.08 (NS)
Lubbock MSA	+23,467	9 %	+133	19 %	+25	9 %	<0.01 (0.09)
McAllen–Edinburg–Mission MSA	+227,001	37 %	+246	41 %	3	3 %	0.62 (NS)
Midland MSA	+15,706	13 %	+33	19 %	+7	5 %	0.21 (NS)
Odessa MSA	+12,405	10 %	+67	36 %	+36	24 %	0.03 (NS)
San Angelo MSA	−473	0 %	+58	27 %	+56	28 %	<0.01 (0.2)
San Antonio MSA	+374,298	21 %	+1,683	47 %	+43	21 %	<0.01 (<0.01)
Sherman–Denison MSA	+8,583	7 %	+92	61 %	+65	49 %	<0.01 (<0.01)
Texarkana MSA	+2,299	3 %	+26	12 %	+22	9 %	0.35 (NS)
Tyler MSA	+33,424	18 %	+193	34 %	+41	13 %	0.02 (NS)
Victoria MSA	+8,382	7 %	−1	0 %	−15	−7 %	0.51 (NS)
Waco MSA	+20,961	10 %	+114	30 %	+32	18 %	0.01 (0.34)
Wichita Falls MSA	−4,796	−3 %	+18	6 %	+18	10 %	0.26 (NS)

**Fig. 2:**
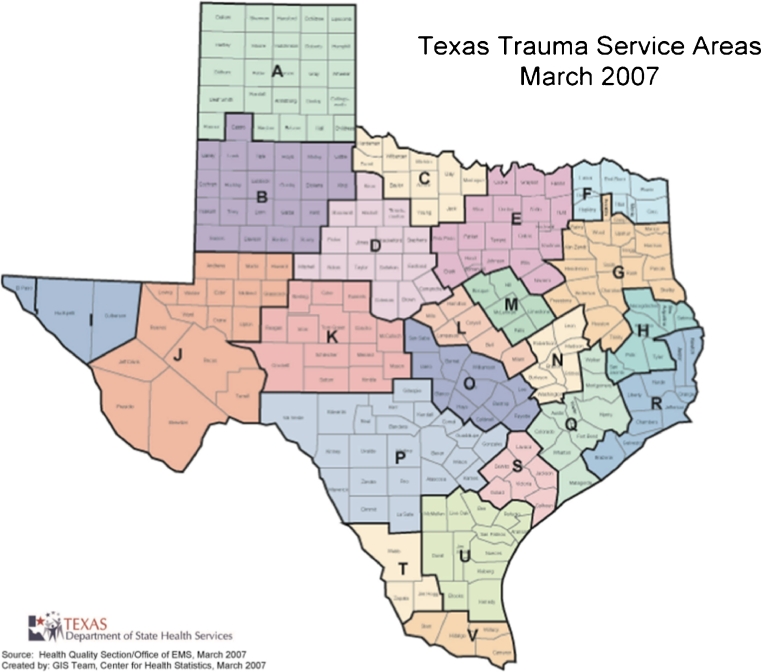
Geographic boundaries and counties in the 22 Texas trauma service areas

**Fig. 3:**
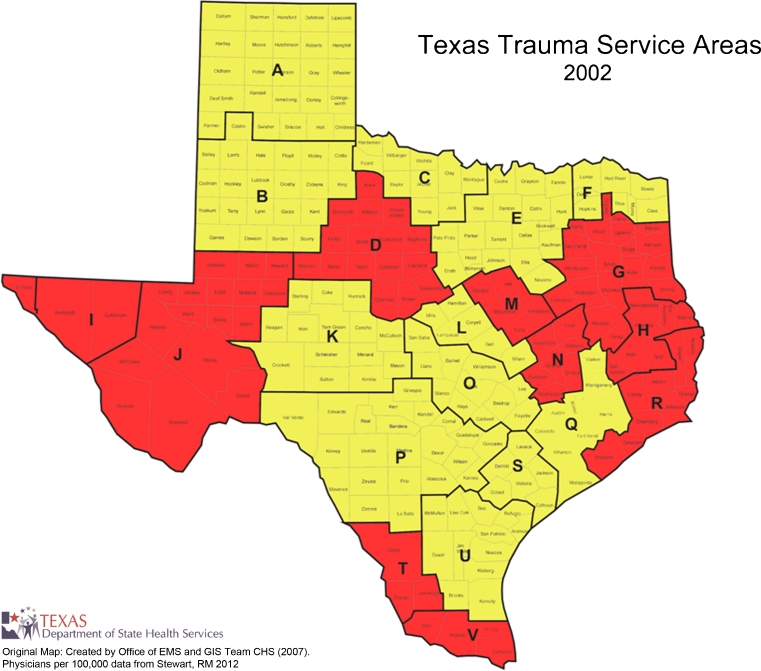
Texas trauma service areas in 2002. Areas with less than 150 physicians per 100,000 residents are shown in red. Those with 150–199 physicians per 100,000 are in yellow, and those with greater than or equal to 200 physicians per 100,000 are in green

**Fig. 4:**
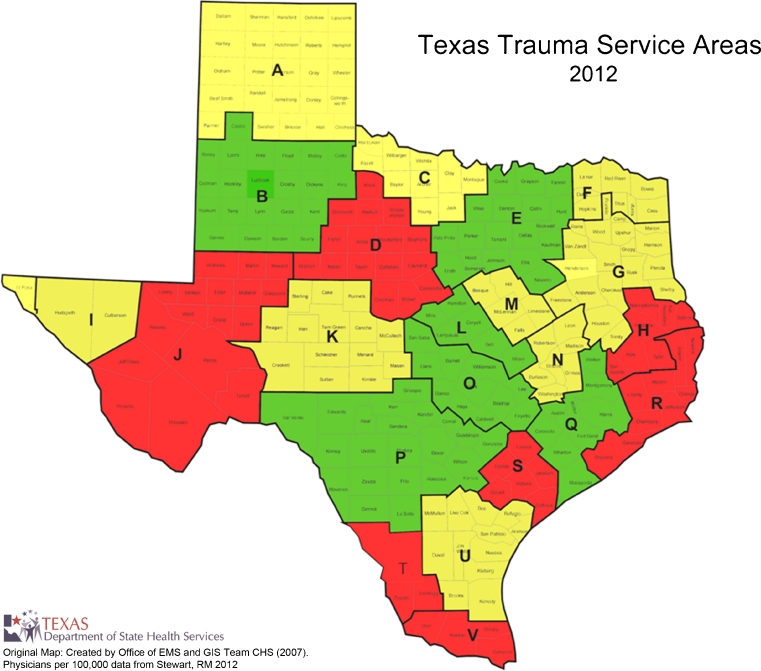
Texas trauma service areas in 2012. Areas with less than 150 physicians per 100,000 residents are shown in red. Those with 150–199 physicians per 100,000 are in yellow, and those with greater than or equal to 200 physicians per 100,000 are in green

TSAs are generally larger than the MSAs. The TSAs include rural and frontier counties, and are intentionally aligned along regional referral patterns (Fig. 2). By geographic area, the largest is TSA-J (West Texas) with 32,447 square miles, while the smallest is TSA-M with 3,884 square miles. By population size, the largest is TSA-E (Dallas–Fort Worth area) with 7.3 million people, while the smallest is TSA-K with a population of 162,047. Table 3 details the changes from 2002 to 2012. There was an increase of 15,599 physicians (44 % absolute increase and an increase of 30 physicians per 100,000 residents). PCPs increased by 6,538 (38 % absolute increase and an increase of 11 PCPs per 100,000; *p* < 0.01). OB-Gyn physicians increased in absolute numbers by 567 (25 % increase, and an increase of 0.3 per 100,000; *p* = 0.28). Surgeons increased by 1,557 (26 % absolute increase, and an increase of 1.1/100,000; p = 0.02). Twenty of the 22 TSAs had an increase in the number of total physicians and physicians per 100,000 population during the period. Figures 3 and 4 display these changes graphically. The greatest increase in physicians relative to the population being served occurred in central Texas. The less populous western and eastern borders of Texas encountered the lowest growth in the number of physicians per capita.

**Table 3:** Change in population, physicians and physicians per capita from 2002 to 2012

Trauma service area	Population change(%)	Total physician change (%)	Per-capita physician change(%)	OB-Gyn change (%)	OB-Gyn per-capita change (%)	PCP change (%)	PCP per-capita change (%)	Surgeon change (%)	Surgeon per-capita change (%)
TSA-A	37,111 (9 %)	113 (19 %)	13 (9 %)	12 (32 %)	1.9 (21 %)	44 (14 %)	3.5 (5 %)	1 (1 %)	−1.9 (−8 %)
TSA-B	29,199 (7 %)	103 (12 %)	10 (5 %)	−6 (−12 %)	−2.0 (−6 %)	−10 (−3 %)	−7.7 (−9 %)	10 (6 %)	0 (0)
TSA-C	−1,272 (−1 %)	31 (9 %)	15 (10 %)	1 (6 %)	0.5 (6 %)	23 (14 %)	10.8 (15 %)	−6 (−10 %)	−2.6 (−10 %)
TSA-D	10,113 (3 %)	32 (8 %)	6 (4 %)	5 (25 %)	1.4 (24 %)	29 (14 %)	7.2 (10 %)	−4 (−5 %)	−2.2 (−8 %)
TSA-E	1,521,282 (26 %)	5,112 (53 %)	35 (21 %)*	201 (29 %)	0.3 (3 %)*	2,133 (47 %)	13 (16 %)*	571 (36 %)	2.1 (8 %)*
TSA-F	11,269 (4 %)	50 (12 %)	12 (7 %)	2 (6 %)	0.2 (2 %)	−12 (−6 %)	−7.5 (−10 %)	5 (6 %)	0.5 (1 %)
TSA-G	96,763 (11 %)	278 (23 %)	15 (10 %)*	4 (6 %)	−0.4 (−5 %)	117 (19 %)	4.9 (7 %)	21 (10 %)	−0.4 (−1 %)
TSA-H	27,221 (11 %)	145 (69 %)	44 (52 %)*	10 (100 %)	3.2 (80 %)	68 (56 %)	20 (41 %)	30 (97 %)	10 (77 %)
TSA-I	91,969 (13 %)	336 (39 %)	28 (23 %)*	27 (42 %)	2.3 (26 %)	180 (45 %)	16 (28 %)*	43 (27 %)	2.8 (13 %)
TSA-J	30,999 (8 %)	89 (19 %)	12 (9 %)	9 (27 %)	1.6 (17 %)	48 (19 %)	6.7 (10 %)	23 (31 %)	4.2 (20 %)
TSA-K	2,462 (2 %)	58 (24 %)	33 (22 %)	0 (0 %)	−0.1 (−2 %)	25 (20 %)	14 (18 %)	3 (6 %)	1.4 (5 %)
TSA-L	70,793 (18 %)	492 (70 %)	79 (45 %)*	5 (15 %)	−0.2 (−3 %)	226 (65 %)	35 (40 %)	68 (65 %)	11 (40 %)*
TSA-M	26,626 (9 %)	118 (27 %)	25 (17 %)*	6 (23 %)	1.1 (13 %)	75 (33 %)	17 (22 %)	5 (6 %)	−0.7 (−2 %)
TSA-N	31,593 (11 %)	142 (37 %)	32 (23 %)*	12 (57 %)	3.1 (41 %)	94 (46 %)	22.9 (31 %)	20 (34 %)	4.3 (21 %)
TSA-O	510,430 (35 %)	1,595 (65 %)	37 (22 %)*	74 (47 %)	0.9 (8 %)	745 (60 %)	16 (18 %)*	184 (48 %)	2.5 (9 %)
TSA-P	404,364 (20 %)	1,711 (43 %)	38 (20 %)*	43 (20 %)	0 (0 %)	640 (36 %)	12 (14 %)*	179 (29 %)	2.3 (8 %)
TSA-Q	1,170,285 (26 %)	4,478 (52 %)	40 (21 %)*	133 (25 %)	−0.1 (−1 %)	1,740 (45 %)	13 (15 %)*	378 (26 %)	0 (0 %)
TSA-R	135,004 (13 %)	264 (18 %)	6 (5 %)	6 (7 %)	−0.5 (−5 %)	111 (15 %)	1.4 (2 %)	18 (8 %)	−0.9 (−4 %)
TSA-S	9,762 (6 %)	−6 (−2 %)	−12 (−8 %)	−2 (−15 %)	−1.6 (−20 %)	8 (6 %)	0 (0 %)	−5 (−13 %)	−4 (−17 %)
TSA-T	64,866 (29 %)	38 (21 %)	−4.5 (−6 %)	3 (18 %)	−0.6 (−9 %)	24 (26 %)	−0.8 (−2 %)	4 (13 %)	−1.8 (−13 %)
TSA-U	23,285 (4 %)	65 (7 %)	4.9 (3 %)	2 (4 %)	0 (0 %)	36 (8 %)	3.0 (4 %)	−14 (−9 %)	−3.5 (−13 %)
TSA-V	319,726 (30 %)	355 (34 %)	2.4 (2 %)	20 (26 %)	−0.2 (−3 %)	194 (31 %)	0.4 (1 %)	23 (14 %)	−2 (−13 %)
Total Texas	4,623,850 (21 %)	15,599 (44 %)	30 (19 %)*	567 (25 %)	0.3 (3 %)	6,538 (38 %)	11.1 (14 %)*	1,557 (26 %)	1.1 (4 %)*

*p < 0.05, chi square with Bonferroni correction for repeated measures

Responses from a hospital survey came from ten health care systems and ten independent hospitals, representing 176 facilities and more than 31,000 licensed beds — approximately 55 % of the private medical–surgical hospitals in Texas. Regarding Question 1 on the impact of reduced liability coverage costs on hospital operations: 58 % of hospitals reported using their reduced liability coverage costs to expand patient safety programs; 51 % reported they used their reduced liability coverage costs to maintain/expand coverage or services for uninsured/underinsured patients; 46 % have used their reduced liability coverage costs to subsidize the various payment shortfalls (e.g., Medicaid); 41 % reported they used the savings to meet monthly obligations, improve salaries for nursing personnel, maintain/increase nurse staffing levels, or maintain/expand staff educational opportunities; 39 % reported using liability savings to maintain/update/add new medical equipment; while 37 % reported using the savings to establish/increase payments to on-call physicians or expand/update their facility. Regarding question (2): Since September 1, 2003 has your facility been able to maintain or expand services (e.g., surgery, primary care, obstetrics)? Sixty-nine percent of the responding hospitals reported that they were able to maintain or expand their services; 60 % reported that they have been able to maintain or expand cardiothoracic surgery, neurosurgery, orthopedic surgery and plastic surgery; 50 % reported that they had been able to maintain or expand their emergency department services; 46 % reported that they have been able to maintain or expand their primary care services; 36 % reported that they have been able to maintain or expand their OB Gyn services; and 33 % reported that they had been able to maintain or expand their general surgery services. Regarding Question 3 — “Since Sept 1, 2003 has your facility been able to maintain or expand its ability to provide emergency or specialized care services (e.g., trauma, surgery) due to a larger number of physicians willing to take call or expand their practice?” — 52 % of the hospitals responded that they have been able to maintain or expand their ability to provide emergency or specialized care services; 65 % responded that they have been able to maintain or expand their ability to provide services due to a larger number of emergency department physicians willing to take call or expand their practice; and 52 % responded that they have been able to maintain or expand their ability to provide services due to a larger number of orthopedic and general surgeons willing to take call or expand their practice; 47 % responded that they maintained or grew services due to a larger number of cardiothoracic surgeons willing to take call or expand their practice; 34 % reported they had been able to maintain or expand their ability to provide services due to a larger number of neonatologists and OB-Gyn physicians; 30 % reported they had been able to maintain or expand their services due to a larger number of neurosurgeons and anesthesiologists willing to take call or expand their practice; and, lastly, 21 % reported they had been able to maintain or expand their ability to provide services due to a larger number of neurologists willing to take call or expand their practice. Regarding Question 4, 80 % of the hospitals indicated that stable/declining *physician* liability insurance costs or a more favorable liability climate had a “significant” or “somewhat significant” impact on the hospital’s ability to provide emergency or specialized care services. For Question 5, 85 % of hospitals indicated that they found it easier to recruit physicians because of either stable/declining physician liability insurance costs or a more favorable liability climate in Texas. Fifty-seven percent of hospitals indicated that they found it easier to recruit OB-Gyn physicians and general surgeons; 50 % found it easier to recruit orthopedic surgeons; 42 % indicated that they found it easier to recruit neurosurgeons; 32 % of hospitals indicated that they found it easier to recruit anesthesiologists and neonatologists; 27 % found it easier to recruit cardiothoracic surgeons and neurologists; and 22 % of hospitals indicated that they found it easier to recruit emergency medicine physicians.

## Discussion

### Key Findings

In this report we have attempted to paint an accurate description of the landscape before and after comprehensive tort reform in Texas. Our data show that the period following tort reform was associated with an increase in the number of physicians relative to the Texas population. This change occurred across most of the regions of Texas, although it was significantly greater in the metropolitan areas and the central geographic areas of the state. Five of the 22 TSAs had increases in the number of physicians in their regions by more than 50 %.

This effect was most prominent among PCPs and surgeons. Physicians practicing obstetrics and gynecology increased in number, but the increase was flat relative to the increase in the Texas population. Of note, there was a 27 % increase in OB Gyn physicians practicing in the nonmetropolitan areas of Texas, although this was not statistically significant.

### Limitations

This report has limitations that should be considered in interpreting these data. As noted above, the rate of physician growth relative to the population was significant; however, these data, from a cross-sectional, prospectively maintained, database which was retrospectively reviewed, do not show causation. There are other factors that may have occurred concomitantly. One of the potentially most significant confounders is the growth in the Texas economy. Texas gross domestic product increased significantly over time, which plausibly could also have influenced the increase in the number of physicians. Although the economy or other factors may have played a role, we believe it is very likely that tort reform had an effect on the disproportionate net increase in physicians. Similar to financial savings, the rate in physician growth does not have to be dramatic to see substantial absolute increases over time. Over the time period of the study medical school class sizes did grow. There was also growth of academic medical centers in Temple, Round Rock, El Paso and Austin. These changes may also have contributed to the increased number of physicians in Texas, potentially independent of tort reform.

### Comparison to Previous Data

Tort reform has been beneficial to Texas physicians. The financial impact of comprehensive medical malpractice tort reform in Texas was soon evident to physicians. Within 1 year of passage of HB-4, the Texas Medical Liability Trust (TMLT), the state’s largest carrier, had reduced its malpractice premiums by 17 %.^14^ The TMLT then cut costs by another 5 % on January 1, 2005. The self-insured University of Texas System Malpractice Plan has reinvested malpractice savings into patient safety and clinical efficacy programs in its academic medical centers. Multiple sources have documented decreased filing of medical malpractice lawsuits. In 2003, there were 1108 medical liability suits filed in Dallas County. This decreased to 142 cases in 2004 and 184 cases in 2005.^5^,^15^ A prior report from our institution showed a 5-fold decrease in the number of malpractice claims and a 55 % reduction in the cost of premiums following tort reform in 2003.^7^ In May 2006, the American Medical Association (AMA) removed Texas from its list of states experiencing a liability crisis, marking the first time the AMA has removed any state from its crisis list.^16^

By 2002, most physicians and health care organizations believed that excess malpractice lawsuits had started to affect the delivery of health care. Most felt that physicians had left or avoided geographic areas of Texas known as “litigious hotbeds,” while others totally avoided high-risk procedures.^2^,^15^ Advocates reported many physicians no longer performed nursing home work and hospitals experienced great difficulty in obtaining physician on-call coverage for emergency rooms, trauma centers, and emergency surgery and obstetrics.^17^

Within 2 years of tort reform, advocates reported that Texas had gained 3,000 physicians, including 93 orthopedic surgeons, 91 obstetrician–gynecologists, 24 neurosurgeons, 20 pediatric cardiologists, 14 pediatric oncologists and ten pediatric surgeons.^2^ These reports were consistent with data that states that have enacted medical malpractice tort reform and capped the non-economic award have had an increased supply of physicians, especially in rural areas.^6^

As of 2011, there appeared to be an influx of practices into the Rio Grande Valley, many in critical medical specialties hardest hit by the liability crisis.^17^ Since 2007, Texas has consistently awarded licenses to 60 % more new physicians each year compared to the 3 years preceding tort reform.^1^,^17^,^18^ Advocacy groups have estimated there have been an additional 6.4 million office visits secondary to tort reform.^8^,^17^,^18^ According to the Department of Health and Human Services, Texas ranks tenth nationally in the percentage growth of patient care physicians per capita, up from 23rd just 5 years earlier.^8^

There is, of course, opposition to comprehensive tort reform in Texas.^9^,^19^ Most opponents are associated with or supported by those in the tort business, but opposition has also come from consumer or public rights advocacy groups. Public Citizen, a not for profit consumer rights group, published a report in October 2011 entitled, “A Failed Experiment: Health Care in Texas Has Worsened in Key Respects Since State Instituted Liability Caps in 2003”.^19^ A segment of this report addressed a putative decline in physicians per capita. The authors opted to use a Texas Department of State Health Services (DSHS) definition of Direct Patient Care Physicians that excludes a number of physician groups including medical school faculty, (DSHS Description: “The reasoning behind making these selections is that only Direct Patient Care Physicians (not Faculty, Researchers, etc.) are actually treating patients as opposed to doing administrative work, teaching, or research…”). From our vantage point, this is, at best, an antiquated notion that excludes very large numbers of practicing physicians from their analysis. As a concrete example, over the past year the faculty of the University of Texas Health Science Center at San Antonio Department of Surgery provided approximately 86,000 patient care visits and performed more than 6,000 operations. A significant portion of this care was for patients with limited or no health care funding, or for patients from rural South Texas. Using the Direct Patient Care Physician definition completely excludes this care. Using a the definition of all active practicing physicians (as reported in this manuscript), leads to data which support a conclusion directly contrary to Public Citizen’s report. With respect to the non-metropolitan areas of the State, our data and that of the Public Citizen are in general agreement: there have been absolute increases in physicians, but no change in physicians per-capita in the post-tort reform period. Hyman, Silver and Black describe an in-depth analysis of physician numbers relative to Texas population.^20^ These authors use a similar methodology (Direct Patient Care Physician) to the Public Citizen, thereby excluding very large numbers of physicians who actually provide a significant amount of direct patient care—physicians employed or associated with a medical school faculty. For the reasons noted, we believe this methodology does not properly account for physicians providing significant patient care. Additionally, the DSHS definition has changed over the time period of the study, making interpretation of data using the Direct Patient Care Physician definition even more difficult. In short, we believe the simpler and more consistent definition of all active practicing Texas physicians is the best way to account for changes in the physician workforce before and after tort reform.

Most of the reports refuting the benefits of tort reform come from non-health care professionals; however, there are also dissenting voices in the medical and surgical community concerning potential benefit for the patient.^21^–^23^ Although we do not generally agree with these critics, we do agree that the cost savings and benefits of tort reform should be passed along to the consumers of health care, not simply the providers of health care, and we believe that we have a professional obligation to see that patient care and service improves.

### Challenges in Texas

Each region in the United States has unique challenges and unique strengths with respect to caring for patients. Texas has a rapidly increasing population, a tremendous degree of geographic and population diversity, and a high percentage of patients without any health care coverage (25 %). These factors create great challenges to improving access to health care, and highlight the need for developing strategies to recruit and retain physicians into Texas. It is highly probable that comprehensive tort reform is one of those strategies that have been successful in improving patients’ access to health care. To be more specific, we do not believe that tort reform is usually the primary factor that leads to individual physician recruitment or retention; however, it is clearly a permissive factor, which likely leads to decision making that greatly facilitates physician recruitment and retention. As the hospital survey data demonstrate, those recruiting physicians have found it significantly easier to do so in the post-tort reform period (85 % of responding hospitals). The second point illustrated by the survey data is that access is more than just absolute number of physicians or physicians per capita, it is also about what services physicians provide. Fifty-two percent of hospitals reported being able to expand emergency or specialized services due to more physician call availability or physicians’ willingness to expand their practices. Eighty percent of hospitals reported they had been able to expand services due to the improved physician liability climate.

## Conclusions

Our data demonstrate the post-tort reform period in Texas was associated with a significantly increased growth rate of physicians relative to the Texas population. The net change was seen across most regions of Texas, although it was significantly greater in the metropolitan areas of Texas and the central geographic areas of the State. These changes led to an expansion of physician and hospital services throughout Texas. We conclude that tort reform, as implemented in Texas, provides a needed framework for improving access to health care.
